# Development of SSR markers and analysis of diversity in Turkish populations of *Brachypodium distachyon*

**DOI:** 10.1186/1471-2229-9-88

**Published:** 2009-07-13

**Authors:** John P Vogel, Metin Tuna, Hikmet Budak, Naxin Huo, Yong Q Gu, Michael A Steinwand

**Affiliations:** 1 USDA-ARS, Western Regional Research Center, Albany, CA, USA; 2 Namik Kemal University, Department of Field Crops, Tekirdag, Turkey; 3 Sabanci University, Biological Science and Bioengineering Program, Istanbul, Turkey

## Abstract

**Background:**

*Brachypodium distachyon *(Brachypodium) is rapidly emerging as a powerful model system to facilitate research aimed at improving grass crops for grain, forage and energy production. To characterize the natural diversity of Brachypodium and provide a valuable new tool to the growing list of resources available to Brachypodium researchers, we created and characterized a large, diverse collection of inbred lines.

**Results:**

We developed 84 inbred lines from eight locations in Turkey. To enable genotypic characterization of this collection, we created 398 SSR markers from BAC end and EST sequences. An analysis of 187 diploid lines from 56 locations with 43 SSR markers showed considerable genotypic diversity. There was some correlation between SSR genotypes and broad geographic regions, but there was also a high level of genotypic diversity at individual locations. Phenotypic analysis of this new germplasm resource revealed considerable variation in flowering time, seed size, and plant architecture. The inbreeding nature of Brachypodium was confirmed by an extremely high level of homozygosity in wild plants and a lack of cross-pollination under laboratory conditions.

**Conclusion:**

Taken together, the inbreeding nature and genotypic diversity observed at individual locations suggest a significant amount of long-distance seed dispersal. The resources developed in this study are freely available to the research community and will facilitate experimental applications based on natural diversity.

## Background

The small grass *Brachypodium distachyon *(Brachypodium) is fast emerging as a powerful model system to study questions unique to the grasses. Brachypodium possesses the physical and genomic attributes (small stature, fast generation time, simple growth conditions, small genome, self-fertile, diploid, annual lifecycle) necessary to be a modern model organism. A truly tractable model grass is needed because the extremely powerful model dicot, *Arabidopsis thaliana*, cannot be used to answer questions where dicot and grass biology diverge (e.g. cell wall composition). Humans derive the majority of their food directly or indirectly from grasses and are projected to use increasing amounts of energy derived from grasses grown as biofuel crops. Thus, there is considerable need for a model grass to facilitate research aimed at improving grasses as grain, forage and energy crops to supply an ever increasing human population with food and energy. The emergence of Brachypodium as a model organism fulfills this need.

Brachypodium is a typical grass in terms of architecture and growth habit [1,2]. Thus, Brachypodium is an excellent functional model for all grasses including the large perennial grasses like switchgrass and *Miscanthus *that are being developed as dedicated energy crops. In this context, the small size (15 cm), compact genome (300 Mb), and rapid generation time (8 weeks) of Brachypodium will facilitate the application of modern high-throughput genomic technologies toward questions relevant to energy crops. It is particularly important to have a model for emerging energy crops because their large size, large genomes and outcrossing nature make the use of many powerful experimental approaches (mutant screens, transgenic manipulation, growth under controlled conditions) difficult in these species. Thus, it is not surprising that the rapid development of numerous tools for Brachypodium is coincident with increased interest in research topics relevant to the development of grasses as biomass crops for the sustainable production of biofuels. One important area of investigation is the unique cell wall of grasses. In this context, Brachypodium is an excellent system because its cell wall is typical of grasses including switchgrass and *Miscanthus *[3]. Thus, knowledge in this area gained from Brachypodium will be applicable to energy crops. Brachypodium is closely related to wheat and the small grains [4-6] and thus, in addition to serving as a functional genomic model, holds promise as a structural genomic tool to aid the exploration of the huge genomes found in these crops. Several studies have already used the closely related species *Brachypodium sylvaticum *as a tool to aid cloning projects in wheat and barley [7-9].

In addition to serving as a traditional model, Brachypodium is an excellent test-bed for transgenic approaches in the grasses because of an extremely efficient *Agrobacterium*-mediated transformation system and a rapid generation time. We currently obtain average transformation efficiencies over 50% for high-throughput production of T-DNA tagged lines (unpublished) and efficiencies ranging from 30–80% have been reported by three groups [10-12]. (see  for a variety of up-to-date protocols). Other experimental resources that have been developed or are under development to enable Brachypodium to be used as a model organism include: identification of growth conditions that allow rapid generation times (as fast as 8 weeks from seed to seed), BAC libraries, physical maps, methods for ethane methyl sulfonate and fast neutron mutagenesis, cDNA libraries, and EST sequences [6,13-15]. In addition, a high-density genetic linkage map has been constructed (unpublished) and, most importantly, is the imminent completion of the whole genome sequence including a large EST sequencing component to aid annotation.

Missing from the list of Brachypodium resources is a large collection of diverse inbred lines and genetic markers. There are currently only six freely available inbred diploid lines [12,15]. Some additional collections have been made, but are available only under a restrictive material transfer agreement [1]. A diverse collection of well described, freely available inbred lines is essential to allow Brachypodium to be used to study natural variation, and to allow positional cloning of induced mutations. Genetic markers are essential for many experiments including positional cloning, mapping quantitative trait loci, association mapping, ECOTILLING and analysis of genotypic diversity in populations. PCR-based markers are particularly useful because they are fast, easy to score and can be used by any lab with standard molecular biology tools. Simple Sequence Repeats (SSRs), also known as microsatellites, are genomic areas with simple short repeat units. The number of repeats in these regions is highly polymorphic and thus markers developed from SSRs are particularly powerful. Both markers and a diverse collection of inbred lines are necessary for Brachypodium to reach its full potential as a model system. We addressed these needs by developing SSR markers and creating inbred lines from a diverse collection of Brachypodium accessions collected from 53 locations throughout Turkey.

## Results

### SSR identification and initial survey of polymorphism

**Figure 1:**
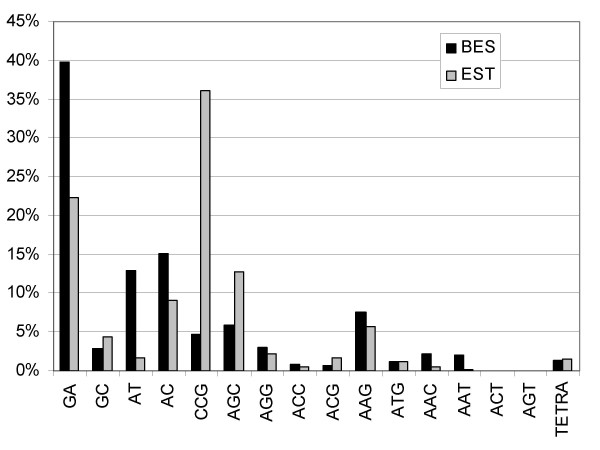
**Distribution of repeat classes found in BES and ESTs**.

In total, 1,166 SSRs were identified (519 from 20,440 ESTs and 647 from 44,952 BES) using cutoff values of six repeats for dinucleotide repeats, five repeats for trinucleotide repeats, and four repeats for tetranucleotide repeats. The most common repeat from the ESTs was the trinucleotide repeat CCG which comprised 36% of all EST-derived SSRs and the most common repeat from the BES was the dinucleotide repeat GA which comprised 40% of all BES-derived repeats (Figure 1). The higher percentage of trinucleotide repeats in the ESTs suggests that many EST SSRs are constrained by being in coding regions.

To identify robust, polymorphic markers for population studies we determined the level of SSR polymorphism in a small collection of six Brachypodium lines (Bd1-1, Bd2-3, Bd3-1 Bd18-1, Bd21, Bd21-3) using 621 primer pairs (144 from ESTs and 477 from BES) (additional file 1). Out of the 621 primer pairs tested, 398 (64%) produced strong bands. Of those, 261 (66%) primer pairs identified polymorphisms between at least two of the six lines. As has been observed in other systems, SSRs with more repeats were more polymorphic than SSRs with fewer repeats. For example, SSRs with five repeat units had an average of 1.5 alleles whereas SSRs with 13 or more repeats had 3.2 alleles. The diversity of SSR alleles found in this initial population indicates that there is sufficient SSR diversity to use SSR markers for mapping or population analysis. Full details of product sizes for all markers are presented in additional file 2.

### Collection of new accessions and creation of inbred lines

**Figure 2:**
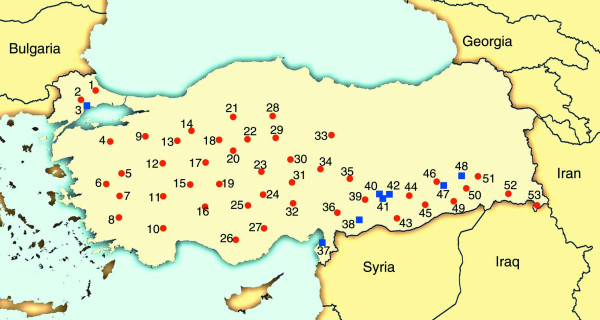
**Location of collection sites in Turkey**. Numbers correspond to the location numbers listed in additional file 3. Collections made by HB are designated by red dots and collections made by MT are designated by blue squares.

Two new collections of Brachypodium accessions were used in this study. The first collection was made by MT between June 6 and July 10, 2006 from eight locations in Turkey (Figure 2). For three locations (Adiyaman, Gaziantep, Tekirdag) an area of ~10,000 m^2 ^was sampled by collecting seed from individual plants scattered across the site. Bulk collections of several plants at different sub-sites were also made. Inbred lines were made from each of the different individual plants that were collected. From five sites (Balli, Bismil, Iskenderun, Kahta, Kozluk) only one bulk collection from plants growing in small area of a few square meters was made. For these locations eight inbred lines were made by sub-sampling the bulk seed. All sites except Tekirdag were overgrazed hilly pasture with continental climate (very dry with hot summers and cold winters). Tekirdag lies near the Marmara Sea and so has a more maritime climate (higher humidity, more precipitation, relatively cooler summer and warmer winters). The Tekirdag site is also forested and so was shaded and not subject to grazing. The 84 inbred lines developed from material collected by MT were named using the first three letters of a nearby town as the prefix. The locations and other details of these collections are summarized in additional file 3. These lines were inbred for two generations.

The second collection was made independently by HB in 2006 from 45 locations across Turkey (Figure 2). Full details of these collections, the development of inbred lines and phenotypic analysis will be published elsewhere. One hundred twenty one inbred diploid lines from this collection were used in the present study (additional file 3). These inbred lines were placed into 13 phenotypic groups and the naming convention consists of the group prefix (e.g. BdTR1) followed by a unique letter for each inbred line. The phenotypic groups BdTR4 and BdTR6 were polyploid and were not included in the present study. For this collection it is important to note that lines with the same prefix (e.g. BdTR1a and BdTR1e) do not necessarily come from the same location (additional file 3). In addition to the new collections, six previously described inbred lines, Bd1-1, Bd2-3, Bd3-1, Bd18-1, Bd21 and Bd21-3 were also used [12,15].

### Phenotypic characterization

For the inbred lines developed from material collected by MT, we examined flowering time, vernalization requirements, seed size and presence of hairs on the lemma. This phenotypic analysis focused on easily scored phenotypes in order to determine if there was major phenotypic diversity in the collection. It is not intended to be an exhaustive examination of phenotype by environment interaction but rather to provide a resource to allow users to select lines with potentially useful variation in traits of interest. Flowering time was highly dependent upon the length of vernalization (additional file 4). All the lines required longer vernalization times to induce flowering than Bd21, Bd21-3, Bd2-3 or Bd3-1. Four or five weeks of vernalization was sufficient to induce rapid flowering in all lines except those from Tekirdag. The lines from Tekirdag required much longer vernalization times from 8–16 weeks. Growth under long day conditions (20 hr light: 4 hr dark) has previously been shown to promote the flowering of Bd21, Bd21-3, Bd2-3 and Bd3-1 in the absence of vernalization [12,15]. However, these long day conditions failed to induce rapid flowering of any of the material collected by MT or HB.

**Figure 3:**
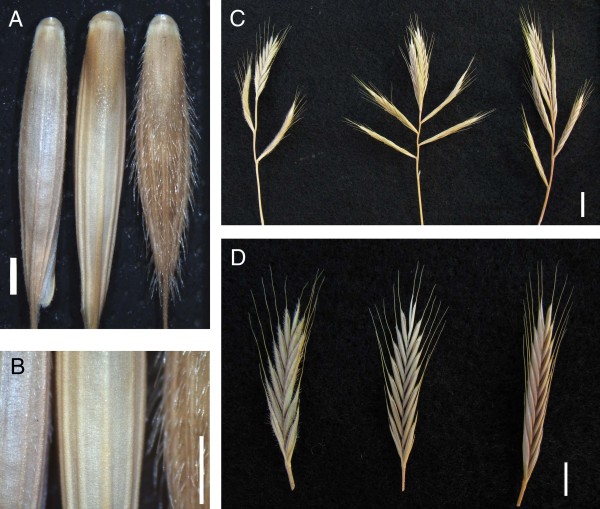
**Phenotypic differences between diploid lines**. (A and B) The presence of hairs on the lemmas differed dramatically. From left to right are lines Tek-1, Koz-3 and Koz-4. Note the presence of a few very short hairs on Tek-1 and absence of hairs on Koz-3. Scale 1 mm. (C and D) Spikes from three lines grown under the same conditions. Note the variation in number of branches, angle of branches, number of seeds per spike and angle of the seeds. From left to right are lines Adi-13, Tek-1 and Tek-4. Scale 1 cm.

Seed size ranged from 2.5 to 5.9 mg/seed for the diploid lines. The average seed size was 4.1 mg/seed (additional file 5). There were significant differences between the average seed sizes from the different populations (ANOVA, p-value = 0.001). Most of this difference was due to the smaller seed size of the lines from Tekirdag. The average seed size of the lines from Tekirdag (3.3 mg/seed) was significantly (t-test, p-value = 0.003) less than the average seeds size of the lines from all other locations (4.3 mg/seed). The lines from Tekirdag had short hairs on their lemmas and appeared smooth whereas most other lines had very hairy lemmas (Figure 3). Bd18-1 seeds had short hairs similar to the Tek lines. Bd18-1 also requires a long vernalization similar to the Tek lines. One line, Koz-3 had no visible hairs on the lemma (Figure 3). We also observed variation in the number of inflorescence branches, number of seeds per branch, and the angle of the seeds and branches (Figure 3). It should be noted that the number of branches produced by a given line is highly dependent upon vernalization. Therefore, only lines grown under the same conditions and vernalized such that they flower at the same size can be compared. Longer vernalization times lead to fewer branches, fewer tillers and lower seed production.

Ploidy was determined by flow cytometry [16]. For the material collected by MT, c-values from representatives of each population were determined first. Later, representative inbred lines were tested to confirm initial results. Five locations contained only diploid accessions, two locations had only polyploid accessions and one location had both diploid and polyploid plants (Table 1). One inbred line, Adi-P1, appeared phenotypically polyploid, but was collected from a location where c-values from population samples indicated diploidy. Flow cytometry of Adi-P1 confirmed that it was indeed polyploid while other inbred lines from Adiyaman were diploid (Table 1).

**Table 1:** Ploidy determination of material collected in this study

line	Location	2c-value	ploidy
Bd21-3	Iraq	0.76	diploid
population samples	Adiyaman	0.69*	diploid
Adi-5	Adiyaman	0.71	diploid
Adi-P1	Adiyaman	1.41	polyploidy
population samples	Balli	1.32*	polyploidy
population samples	Bismil	0.7*	diploid
population samples	Gaziantep	0.69*	diploid
population samples	Iskenderun	1.31*	polyploidy
population samples	Kahta	0.7*	diploid
Kah-2	Kahta	0.72	diploid
population samples	Kozluk	0.68*	diploid
population samples	Tekirdag	0.7*	diploid

* The value presented is the average of 4–10 c-values obtained from individual plants.

**Figure 4:**
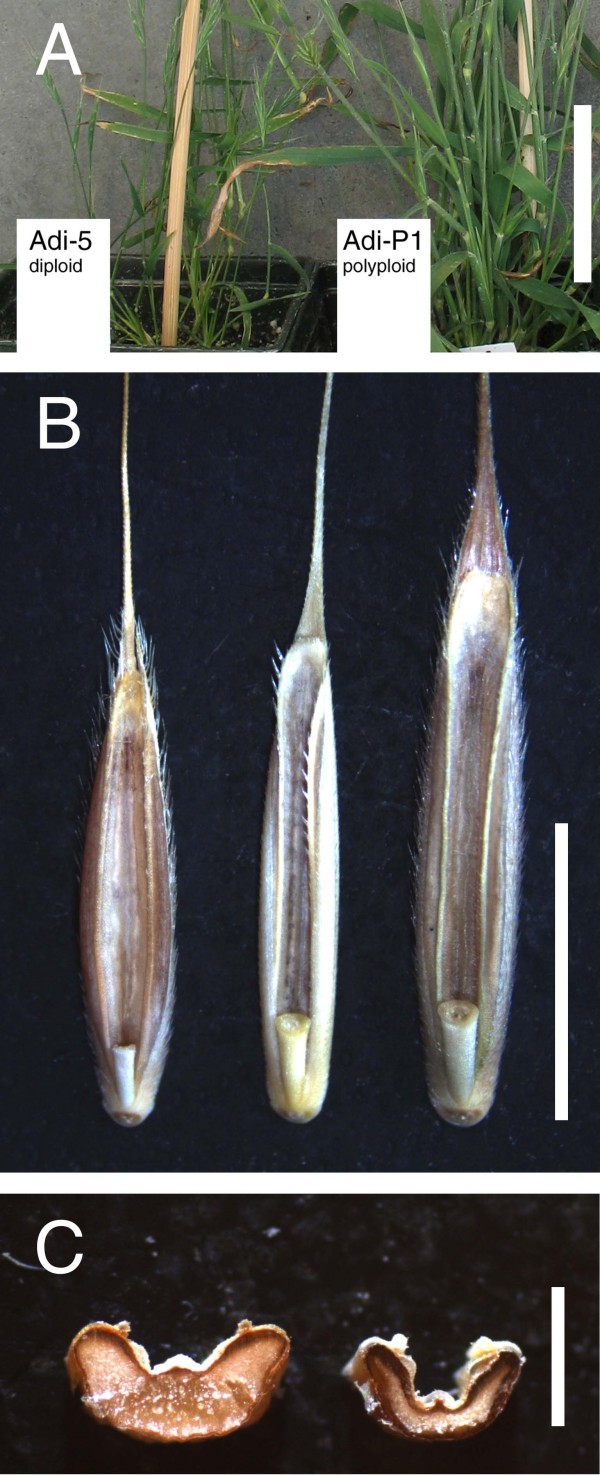
**Phenotypic characteristics of polyploid lines**. (A) Stems of the diploid line Adi-5 and polyploid line Adi-P1. Note the thicker, hairier stems on Adi-P1. Scale is 5 cm. (B) Seeds of, from left to right, Adi-5, Isk-P1 and Adi-PI. Note the larger size of Adi-P1 and deeper crease of Isk-P1 as compared to Adi-5. Scale is 5 mm. (C) Cross section of Adi-5 (left) and Isk-P1 (right) showing the deep crease in Isk-P1. Scale is 1 mm.

While ploidy level in some grass species (e.g. buffalograss) cannot be distinguished visually [17], in Brachypodium when grown side by side polyploid lines were easily distinguished from diploid lines by visual examination (Figure 4). Two forms of polyploids were apparent. One form typified by Adi-P1, lines from Balli, and BdTR6 lines has much larger seeds. For example, Adi-P1 has an average seed size of 10.1 mg, much larger than the 4.1 mg/seed average for all diploid lines (additional file 5). This form also had much thicker and hairier stems (Figure 4). The other form typified by lines from Iskenderun and BdTR4 lines had seeds similar in size to diploid seeds, but with a more pronounced crease (Figure 4). These polyploid lines also tended to bear more seeds per spike, though this trait is variable depending upon the conditions. Another difference between polyploid and diploid lines is the degree to which anthers exert. Anthers from flowers of both polyploid forms often exert whereas, under our conditions, anthers rarely exert from diploid flowers.

### SSR analysis and inbreeding

Based on our initial survey of SSR sizes in six inbred Bd_lines, we selected 43 SSR markers that produced robust bands and were highly polymorphic among the Bd_collection to genotype the entire collection (Table 2). The number of alleles per marker ranged from three to 24 to and the average number of alleles was 10.2 (Table 2). Summary statistics for the markers are presented in additional file 6 and all the SSR sizes are presented in additional file 7. Out of the 8,041 genotypes determined, only four heterozygous plants that were each heterozygous for one marker were found. This indicates that the lines are highly inbred. To determine the prevalence of Brachypodium self pollination in the wild, for 62 of the lines we genotyped the first plant to be grown under greenhouse conditions. Since these plants had undergone no inbreeding in the lab they are representative of wild plants. These 'wild' plants were overwhelmingly homozygous. In fact, only one marker from one plant was heterozygous. There was an average of 1.9 alleles per marker per population in this sample. Therefore, if there was a significant amount of outcrossing, we would have seen many more heterozygous individuals. This indicates that Brachypodium is primarily a selfing species under field conditions. In further support of Brachypodium's inbreeding nature, we have observed that diploid flowers rarely open under our greenhouse and growth chamber conditions. Under certain environmental conditions (warm and humid in full sun) we have observed open flowers on diploid plants grown outside. However, inspection of these open flowers revealed that the anthers had already dehisced on the stigmas under the fold of the palea. Thus, even open flowers are expected to produce an overwhelming proportion of self pollinations. Due to the high degree of homozygosity we stopped inbreeding the lines developed from MT collections after only two single seed descent generations under laboratory conditions.

**Table 2:** Markers used to survey population diversity*

Marker name	Reverse primer	Forward primer (includes M13 tail)	Repeat unit	No. of Repeats	No. of alleles in six Bd lines	No. of alleles in all lines
ALB001	ttctccaccacactccttcc	cacgacgttgtaaaacgacgatcgtgttcttccccttca	ct	22	4	12
ALB006	cctcctccaacaaccacagt	cacgacgttgtaaaacgactagtagcccccaagccttct	gt	15	4	10
ALB008	atgggcgagaagacagagaa	cacgacgttgtaaaacgacacgggaactcaccatttcac	ctt	13	4	11
ALB013	atccgtgtttcgttctttgg	cacgacgttgtaaaacgactttgccaatgcttcaaactg	ga	12	3	9
ALB022	atttctgcccagcaaacact	cacgacgttgtaaaacgacgacctcccctgtctcaggt	ct	11	3	12
ALB030	gtagtccagccccatcttcc	cacgacgttgtaaaacgaccgtccgcaagcttgtttt	ttg	9	3	16
ALB034	tgctgcttcttttgtcgatg	cacgacgttgtaaaacgacaatggtggatctgcagaagg	aac	9	3	7
ALB040	agtcctcctcctcgctaagg	cacgacgttgtaaaacgacccctgcttccctctctctct	ctt	8	3	10
ALB050	ggagggagaaaaatgccttc	cacgacgttgtaaaacgactagtagcccccaagccttct	gt	15	4	11
ALB056	tccacagagcaccacagaag	cacgacgttgtaaaacgactcctctggttcctgagatcg	gt	10	3	9
ALB086	caactgatcccgagctcttc	cacgacgttgtaaaacgacgtccggaaccaacgaaaac	aag	7	3	7
ALB087	gacttgatgaagccctgctc	cacgacgttgtaaaacgacacaggcagcagcaggaac	agc	7	3	6
ALB089	tgagtcgaataagccggaag	cacgacgttgtaaaacgaccttcacccagctgctcatc	cag	7	3	8
ALB100	caggtacgtcaccaggttca	cacgacgttgtaaaacgaccggagacgacgacagagg	gca	7	3	14
ALB131	gacacatcgttggcaatgtc	cacgacgttgtaaaacgaccaacggagtggtacgttgtg	ggc	7	4	7
ALB139	tgtaccggaggatgaagtcc	cacgacgttgtaaaacgacgtgccaaatccaagaaggaa	aga	7	3	11
ALB155	atatcacccccacaggaacc	cacgacgttgtaaaacgactgatcagagtccccaaaacc	ct	15	3	6
ALB158	tgtcttgtcccctgcgata	cacgacgttgtaaaacgacgcgctccttgagctgtattg	gaa	14	4	14
ALB160	tgctgatcccatttcctcat	cacgacgttgtaaaacgacgtgcccgaggactatcatgt	ct	13	3	10
ALB165	atttgccccacaaatggtta	cacgacgttgtaaaacgacttcgtggttcaacaacatgg	ata	12	3	18
ALB170	agggtggctgtttagacgaa	cacgacgttgtaaaacgacttgggaatctccaggttcag	tg	10	3	15
ALB175	ctgacgttagggtggctgtt	cacgacgttgtaaaacgacttgggaatctccaggttcag	tg	10	4	9
ALB179	caccggaagtggagaagaag	cacgacgttgtaaaacgactgatcaagtgcaaggaaacg	ct	9	4	24
ALB181	gggttccacctgtcagaaaa	cacgacgttgtaaaacgacatgccaaatgggactgtttc	ac	9	3	6
ALB183	tggtgatttaaatggcacaaa	cacgacgttgtaaaacgacgggatatgcccaattttgaa	tct	9	3	7
ALB223	aagacatccaaccgaccaag	cacgacgttgtaaaacgacactgctgcgtttcgtcaga	aat	6	3	7
ALB230	taaaggggcaaattgcaaag	cacgacgttgtaaaacgactcgcaatgaaaccctaggtc	atct	6	3	13
ALB257	tgtgatggattttgcttcca	cacgacgttgtaaaacgactgtcgctcctgcatctattg	aatt	5	3	10
ALB273	tccatctccatcatcccttc	cacgacgttgtaaaacgactggggaacatttccatcatt	ga	8	3	4
ALB278	cctgggttaattatggcctgt	cacgacgttgtaaaacgaccgtatctattctcacctccgatg	tc	8	3	5
ALB311	cgtcgtcttcaggtctttcc	cacgacgttgtaaaacgaccctaacagcttccgtctcca	ga	6	3	9
ALB348	tgtgtgccgaactagtgaaag	cacgacgttgtaaaacgactccaggccctcacatatctc	tc	13	4	13
ALB349	ggatggctctcaaggtcact	tcacgacgttgtaaaacgacgagcatgtgggtgtgattt	tc	13	5	16
ALB355	cagcaggtcctcgtactcct	cacgacgttgtaaaacgacgatacatcccagccattaatcc	ctt	11	4	16
ALB372	ggtgcgcaatggagatagat	cacgacgttgtaaaacgactctaggctccgttccgagta	tatc	9	4	13
ALB374	aatggtggatctgcagaagg	cacgacgttgtaaaacgactgctgcttcttttgtcgatg	gtt	9	3	7
ALB376	gggttccacctgtcagaaaa	cacgacgttgtaaaacgacatgccaaatgggactgtttc	ac	9	3	6
ALB445	caccagcgtttacgtagcag	cacgacgttgtaaaacgactgttcagtgggtcgagtcaa	gaaa	7	3	11
ALB454	cttcacccagctgctcatc	cacgacgttgtaaaacgactgagtcgaataagccggaag	tgc	7	3	9
ALB461	gggccgtctgcatttatcat	cacgacgttgtaaaacgaccagaactgtcagtcccctttg	at	7	3	6
ALB467	ggcgtcacgaaagaagagaa	cacgacgttgtaaaacgacccccaccagctctatgaaat	at	7	3	6
ALB486	ggccagccatgttagactgt	cacgacgttgtaaaacgactaatccgcgtcctctcttgt	ag	6	3	3
ALB514	aaaagaaccccgacctgaat	cacgacgttgtaaaacgacacggagggagtacaccacaa	aag	6	3	15
					Avg = 3.3	Avg = 10.2

* PCR product sizes for all lines can be found in additional file 7.

To determine the rate of outcrossing under laboratory conditions, we monitored the rate at which pollen from transgenic plants containing a constitutively expressed GUS reporter gene pollinated wild-type plants. To maximize the chance for cross-pollination, we surrounded single wild-type plants with 10–20 transgenic plants in a single 15 cm pot and tied the plants together as they grew in order to bring the flowers into close proximity. To determine how often transgenic pollen fertilized non-transgenic ovules, we scored the progeny of the wild-type plants for expression of the GUS reporter gene. Out of 2,233 progeny (1,494 from growth chamber grown plants and 739 from greenhouse grown plants) from 25 wild-type plants none expressed GUS. This indicates that the rate of outcrossing under 'worst case' laboratory conditions is exceedingly small.

**Figure 5:**
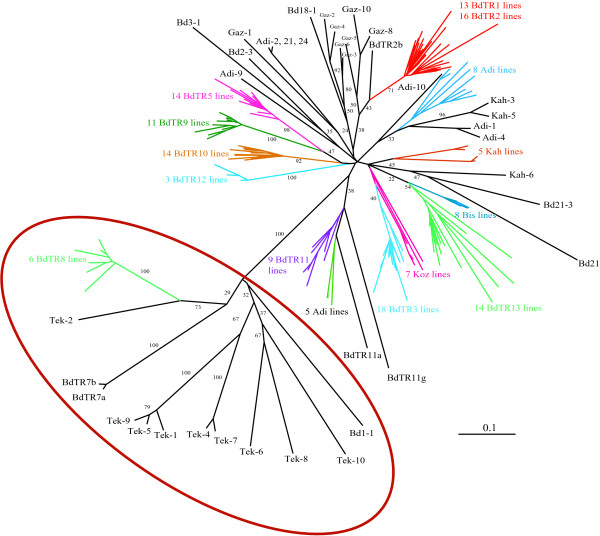
**Phylogenetic analysis**. Unrooted neighbor joining consensus tree of 187 lines based on 100 shared allele bootstrap trees constructed using 43 SSR markers. Bootstrap values greater than 20 are presented for major branches. For clarity, where several lines formed a tight cluster only the number of lines and color-coded line prefixes are displayed. Note that the lines fall into two main groups supported by very high bootstrap support. The lines in the red circle share a number of phenotypic characters (small seeds, hairless lemmas, long vernalization requirements). The other group contains all the other lines from Turkey and Iraq. The major branches in this group have little bootstrap support. Smaller branches with good bootstrap support group the BdTR lines. Additional file 8 shows the same tree as a rectangular phylogram in which all line labels can be read.

To determine the relationship between inbred lines we constructed a consensus neighbor-joining tree based on 100 shared allele bootstrap trees. An examination of the tree reveals two main groups with high bootstrap support (Figure 5 and additional file 8). One group consists of lines from Tekirdag, Bd1-1 and the BdTR7 and BdTR8 groups. These lines are also naturally grouped by their smaller seeds, longer vernalization requirements and nearly hairless lemmas (additional file 4 and additional file 5). The other group consists of all the other lines from Turkey and Iraq. This large group branches into smaller groups, however there is little bootstrap support for the larger branches. There are, however, smaller subgroups with very high bootstrap support. One striking feature is that the 11 BdTR groupings form very tight clusters despite being collected from different locations. Conversely, lines collected from Adiyaman, Gaziantep and Kahta fall into several different groups. In addition to the shared allele tree presented, we constructed several other trees using the other functions in PowerMarker. In all cases, the two main groups and small subgroups with good bootstrap support were apparent (not shown).

## Discussion

The successful implementation of Brachypodium as a model for the study of natural diversity and the positional cloning of induced mutations is dependent upon the phenotypic and genotypic diversity found within the species. Brachypodium occupies a variety of habitats including hot interior regions, cooler coastal areas and colder mountainous regions suggesting the existence of considerable genetic diversity. The natural range of Brachypodium is centered around the Mediterranean extending north into Europe and south into the Indian subcontinent [2]. Within this region, Turkey is expected to be a rich source of Brachypodium diversity because it covers all the habitats mentioned above. Therefore, we have sampled this region heavily (Figure 2). Our results clearly indicate that there is sufficient phenotypic and genotypic diversity within this Brachypodium collection to facilitate studies of natural diversity and allow efficient positional cloning of induced mutations. We had no difficulties identifying polymorphic SSR markers and noted significant differences in easily scored phenotypes like seed size, vernalization requirements, inflorescence architecture and the presence of hairs. This agrees with other groups who have noted diversity in various traits including disease resistance [1,18].

The extremely high degree of homozygosity observed in wild Brachypodium plants indicates that Brachypodium is primarily a selfing species. This is not surprising because as we and others have observed, pollination typically occurs in a closed flower [19]. From a practical perspective, inbreeding simplifies the maintenance of pure lines under laboratory conditions. Indeed, our analysis of pollen flow suggests that even under a 'worst case' scenario it is easy to maintain pure lines. It also means that wild collections do not need to be inbred for many generations to achieve a high degree of homozygosity.

The consensus tree based on SSR polymorphisms clearly shows two major groups with very high bootstrap support (Figure 5). The smaller group containing the Tek lines shared several phenotypic traits including: long vernalization requirements, seed size, and near absence of hairs on the lemmas. The larger group contained lines from Turkey and Iraq. Within this group were tight clusters that contained all the lines from the different BdTR groups that were previously grouped together based on phenotypic similarity. Thus, there was remarkable correlation between the phenotypic groupings and the SSR profiles. Two lines of particular interest, Bd21 the line whose genome was sequenced and Bd21-3 a line that is efficiently transformed were found to be more closely related to one another than to any other line, but they are clearly distinct. The diversity of homozygous genotypes found within very small areas suggests that considerable long-distance seed dispersal takes place. This is consistent with the presence of hairs on the firmly attached lemmas of most lines. These hairs aid seed dispersal by animals and humans. The lack of bootstrap support for the major branches in the largest group suggests that, despite being primarily inbreeding, there is substantial genetic exchange over longer timescales.

## Conclusion

In this study we have demonstrated that considerable genotypic and phenotypic variation exists within this Brachypodium collection. This diversity will allow scientific methods that exploit natural diversity to be applied to Brachypodium. The geographic distribution of SSR genotypes suggests that long-distance seed dispersal plays a significant role in the population structure of Brachypodium. The SSR markers and inbred lines developed in this study are a significant contribution to the Brachypodium resources already available and are freely available to the scientific community.

## Methods

### Identification of SSRs and primer design

SSRs were identified and flanking primers designed essentially as previously described [20]. Briefly, 20,440 ESTs and 44,952 BES generated previously [4,6,13] were analyzed with SSRIT to identify SSRs with at least six unit repeats for dinucleotide repeats, five unit repeats for trinucleotide repeats and four unit repeats for tetranucleotide repeats [21]. Primers flanking the SSRs were designed using BatchPrimer 3 [22]. An M13 primer sequence was added to the forward primer to allow detection with a common fluorescently labeled (VIC or FAM) M13 primer as previously described [20].

### DNA extraction and fragment size detection

DNA was extracted from approximately 0.5 g of leaf tissue as described [23]. The polymerase chain reactions (PCR) were carried out in a final volume of 7.5 μl on an MJ Research PTC-225 thermocycler with a thermal profile consisting of a 2-min initial denaturation step at 95°C followed by 35 cycles of 20 s at 95°C, 20 s at 54°C and 1 min at 72°C. A final 72°C extension step of 30 min was included to promote non-templated nucleotide addition at the 3'end of the PCR product. Reactions were carried out in 10 mM Tris-HCl (pH 9.0 at 25°C), 50 mM KCl, 0.1% (v/v) Triton X-100, 2.5 mM MgCl_2_, 0.1% BSA, 1% PVP-40, 200 μM dNTPs in the presence of 0.5 U *Taq *polymerase, 11.25 ng genomic DNA, 0.5 μM marker-specific reverse primer, 0.033 μM markers-specific M13-tailed forward primer and 0.5 μm VIC- or FAM-labeled M13 primer. PCR products were precipitated with PEG and run on an ABI 3730xl along with PET-labeled size standards of 133–433 bp as described [20].

### Plant growth conditions and phenotypic analysis

Plants were grown in both growth chambers and a greenhouse as previously described [12]. Briefly, for growth chamber experiments the conditions were 20 hr light: 4 hr dark photoperiod with cool-white fluorescent lighting at a level of 150 μEm^-2^s^-1^. For greenhouse experiments there was no shading, temperature range was 24°C in the day and 18°C at night with supplemental lighting to extend daylength to 16 hours.

Seeds were sown in a soilless mix (supersoil, Rod McLellan Co., Marysville, OH) and fertilized once at planting with a time release fertilizer containing micronutrients (Osmocote Plus 15-9-12, Scotts Co., Marysville, OH). To test the effect of vernalization times on flowering, planted pots were placed at 4°C for the desired number of weeks (1, 2, 3, 4, 5 or 16 weeks). While in the cold, pots were continuously illuminated by cool white fluorescent lamps. The vernalization experiment was conducted once in a greenhouse and once in a growth chamber. For each treatment there were six plants in each pot and one pot for each genotype. The Tek lines, were only subjected to 16 weeks of vernalization because preliminary trials indicated that the plants did not flower after 8 weeks of vernalization.

### Pollen flow analysis

To determine the rate of pollen flow under growth chamber and greenhouse conditions, we monitored cross pollination between transgenic and non-transgenic Brachypodium plants. Single wild-type Bd21-3 plants were planted in the center of 15 cm pots and surrounded by 10–20 transgenic Bd21-3 plants. To maximize the potential for pollen flow the plants were tied together such that the flowers from the transgenic plants were touching the wild-type plants. The transgenic line was homozygous for a pOL001 T-DNA insertion that segregated as a single genetic locus. pOL001 contains a GUS reporter gene driven by a constitutive maize *ubiquitin *promoter [15]. The seeds produced by the wild-type plant were planted and the seedlings tested for GUS activity by histochemical staining.

### Phylogenetic analysis

Phylogenetic trees were constructed using several different functions (i.e. shared allele, log shared allele, Euclidean, Reynolds 1983, Nei 1973, Nei 1983, Goldstein 1995, Shriver 1995) within the PowerMarker program [24]. Bootstrapping was carried out using the bootstrap function in PowerMarker and consensus trees were created using the consense program found the PHYLIP software package v3.68 . Frequency based distances were assigned to the consensus tree using FITCH (in PHYLIP package). The neighbor-joining shared allele consensus tree was then edited for presentation using Baobab [25] and Adobe Illustrator (Adobe Systems, San Jose, CA).

## Abbreviations

EST: expressed sequence tag; BAC: bacterial artificial chromosome; BES: BAC end sequence; SSR: simple sequence repeat; PCR: polymerase chain reaction.

## Authors' contributions

JV conceived and designed the study, performed data analysis, made inbred lines, did phenotypic characterization and helped to draft the manuscript. MT made Brachypodium collections and did c-value determinations. HB made Brachypodium collections, made inbred lines, did phenotypic characterization and helped to draft the manuscript. NH and YG provided BES and identified SSRs. MS performed genotyping and helped to draft the manuscript. All authors read and approved the final manuscript.

## Supplementary Material

Additional file 1 **all primers used**. Table of all primers used in this study. Click here for file

Additional file 2 **amplified SSRs with sizes**. SSR size data for Bd lines. Click here for file

Additional file 3 **all lines used in study**. Collection details for all lines used in this study. Click here for file

Additional file 4 **flowering times**. Detail of flowering time experiments. Click here for file

Additional file 5 **seed sizes and pubescence**. Table of seeds sizes and hairiness of inbred lines. Click here for file

Additional file 6 **marker summary statistics**. Table of statistics for markers used in the population survey. Click here for file

Additional file 7 **SSR product sizes used for population analysis**. Table of SSR product sizes used for population analysis. Click here for file

Additional file 8 **phylogenetic tree**. Phylogenetic tree where labels for all lines can be read. Click here for file
