# Supplementary figures and images for: Development of SSR markers and analysis of diversity in Turkish populations of Brachypodium distachyon
Source: BMC Plant Biol. 2009 Jul 13;9:88. doi: 10.1186/1471-2229-9-88 (PMC2719641; doi:10.1186/1471-2229-9-88)

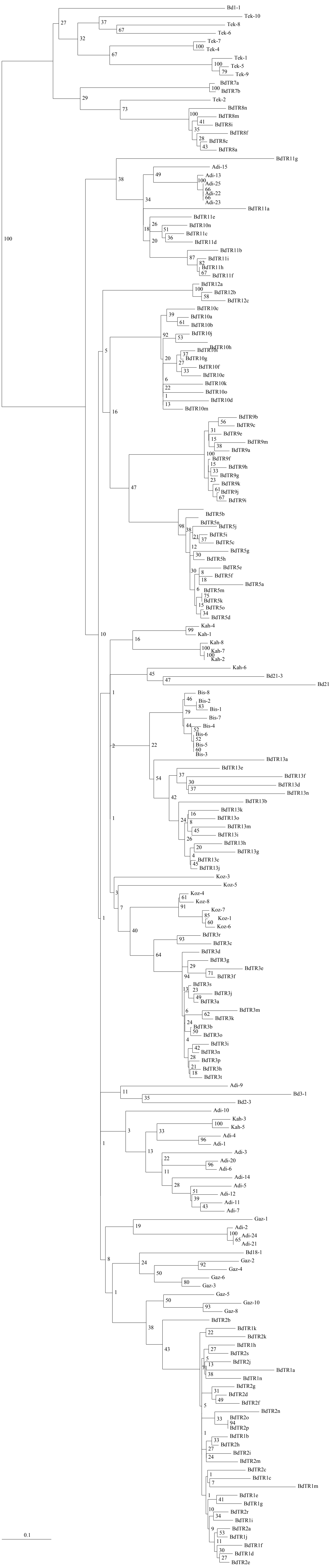

0.1

Supplement: Additional file 8 — phylogenetic tree. Phylogenetic tree where labels for all lines can be read. [file 1471-2229-9-88-S8.pdf]
